# Potential Health Risks of Macro- and Microelements in Commercial Medicinal Plants Used to Treatment of Diabetes

**DOI:** 10.1155/2021/6678931

**Published:** 2021-03-31

**Authors:** Igor D. de Souza, Elaine S. P. Melo, Valdir Aragão Nascimento, Hugo S. Pereira, Kassia R. N. Silva, Paulo R. Espindola, Paula F. S. Tschinkel, Eliza M. Ramos, Francisco J. M. Reis, Iara B. Ramos, Fernanda G. Paula, Karla R. W. Oliveira, Cleberson D. Lima, Ângela A. Nunes, Valter Aragão do Nascimento

**Affiliations:** ^1^Group of Spectroscopy and Bioinformatics Applied to Biodiversity and Health, School of Medicine, Postgraduation Program in Health and Development in the Midwest Region, Faculty of Medicine, Federal University of Mato Grosso do Sul, Campo Grande, Mato Grosso do Sul 79070-900, Brazil; ^2^Institute of Chemistry of the Federal University of Mato Grosso do Sul, Brazil; ^3^Postgraduation Program in Health and Development in the Midwest Region, Faculty of Medicine, Federal University of Mato Grosso do Sul, Campo Grande, Mato Grosso do Sul 79070-900, Brazil; ^4^Centro de Ortopedia e Traumatologia e Medicina do Esporte, Campo Grande, MS, 79021-250, Brazil; ^5^Postdoctoral Student in Program in Biotechnology, Universidade Católica Dom Bosco, Campo Grande, MS 79117-900, Brazil

## Abstract

Information on the content of medicinal plants used in the treatment of diabetes is scarce in the literature. The objectives of this study were to determine the levels of macroelements and microelements in three different medicinal plant species including the dry samples and teas from *Bauhinia forficata*, *Eleusine Indica*, and *Orthosiphon stamineus* and assess the human health risks of ingestion of the tea. The content of the dry samples and teas was obtained using the technique of inductively coupled plasma optical emission spectrometry (ICP OES) after microwave digestion procedure. The hazard quotient (HQ) method was used to access the human health risks posed by heavy metal through tea consumption. The results revealed the presence of K, Mg, Na, P, Al, Fe, Zn, Mn, Cu, Ni, and Se in dry samples and plant teas. The dry plants have high concentration of K and P. All dry plants contain Mg, Na, Al, Fe, Mn, Ni, Zn, and Cu above the limit permissible level set by the World Health Organization (WHO). All the hazard index (HI) values in plant teas were found to be within safe limits for human consumption (HI < 1). The plants may have possible action benefits when used in popular medicine. However, the ingestion through capsules prepared by enclosing a plant powder or teas can be harmful to the health of diabetics. The prescription of this plant for the treatment of diabetes should be treated with caution.

## 1. Introduction

Diabetes is a chronic disease which leads over time to serious damage to the heart, blood vessels, eyes, kidneys, nerves, and poor metabolic control [1]. According to an estimate [2], about 463 million people worldwide in 2019 have diabetes, rising to 578 million by 2030 and 700 million by 2045. According to the World Health Organization, the countries with the lowest economic capacity, the lowest generation, and the distribution of income are those with the highest rates of diabetes prevalence [3]. People with type diabetes must take insulin; however, there is a globally agreed target to halt the rise in diabetes and other diseases by 2025 [4].

There is an alternative therapy useful in the management of diabetes [5]. However, medicinal plants are used to treat diabetes as they are recognized to contain active principles with desired properties [6]. According to published studies, some medical plants are more accessible than conventional medicines and have less side effects compared to synthetic drugs, and they are more effective in the treatment of diabetes mellitus [7]. Thus, many people prefer using them. However, medicinal plant–medicine interactions affect the pharmacodynamics activities of medicines, leading to therapeutic failure or toxicities [8]. In fact, there is a dire need to know the role of action of various medicinal plants and to determine their effect in the therapy of diabetic complications [9].

Medicinal plants used to treat diabetes have high concentrations of K, Ca, Cr, Mn, Cu, and Zn that stimulate the action of insulin [10] and also Fe, Zn, and Cr that act in the prevention of complications of type 2 diabetes [11]. In fact, the macro- and microelements (K, Ca, Mg, Na, Fe, Rb, Sr, Zn, Cu, and Se) play an important role in growth, bone health, fluid balance, and several other processes when ingested in adequate amounts [12–14]. Therefore, prolonged ingestion is high in metals such as Be, Al, Cr, Mn, Fe, Co, Ni, Cu, Zn, As, Se, Mo, Ag, Cd, Sn, Sb, Ba, Hg, Ti, and Pb which can cause toxicity in humans and even death, especially in children [15–17].

In India and Brazilian folk medicine, several plants are used for the treatment of diabetes [18, 19]. In fact, the *Bauhinia forficata* Link. Leaf tea (*B. forficata*) [20], a cerrado native plant commonly known as “Pata de Vaca” (cow's paw), is used as a complementary treatment for diabetes mellitus in Brazil [21–23]. According to the results of studies with this plant, it has prominent potential to combat hyperglycemia [24]. *B. forficata* is native to South America, measuring between 4 m and 12 m in height [25].

In Nigeria, the medicinal plant *Eleusine indica* (*E. indica*) is also used to treat diabetes [26]. The antidiabetic activity of ethanolic leaf extract of *E. indica* was investigated in a model of alloxan-induced diabetes in rats which showed that there are reduced blood glucose levels [26]. On the other hand, in Brazil, aqueous preparations of the grass *E. indica* are used for treating malaria and lung infections [27]. In Malaysia, *E. indica* is traditionally used in ailments associated with the liver and kidneys [28]. *E. indica* is a species of grass that belongs to the family Poaceae; it has about 10 cm to 1 m in height [29].

As discussed earlier in this paper, medicinal plants used as antidiabetic have been investigated for their beneficial health effects. In Malaysia, a search using *Orthosiphon stamineus* (*O. stamineus*) showed that this plant has potential antidiabetic drug in maternal hyperglycemia in streptozotocin-induced diabetic rats [30, 31]. *O. stamineus* is commonly known as “Java tea,” and it has been used in traditional medicine in East India, Indo China, d South East Asia, and Brazil as antidiabetic [32, 33]. *O. stamineus* also known as *Orthosiphon aristatus* (*O. aristatus*) is a herb that is widely grown in tropical areas as Brazil and other countries. *O. aristatus* can be identified by its white or purple flowers bearing long, protruding stamens that resemble cats' whiskers and stem reaching a height ranging from 0.3 to 1 m [34].

In several countries, the medicinal plants *B. forficata*, *E. indica*, and *O. stamineus* have been reported to be useful in diabetes worldwide and have been used empirically in antidiabetic and antihyperlipidemic remedies; however, there are no studies on the quantification of macro- and microelements in these plants. To the best of our knowledge, there are no scientific reports available on the human health risk assessment of some metals and nonmetals in these plants.

The processing and prescription of medicinal plants in capsules, pills, or teas have been common in several countries [35–37]. However, the presence of metals and nonmetals in plants can cause damage to the health of patients. In fact, some plants can interact with prescription or over-the-counter medicines. Thus, it is necessary that only health professionals prescribe medicinal herbs and that there is a quality control.

Motivated by the manuscript published by Tschinkel et al. [38], which emphasizes the need for strict control of the presence of chemical elements and dosage labeling, the objective of this study was to determine the concentration of macroelements (K, Mg, Na, and P) and microelements (Al, Mn, Co, Cu, Ni, Fe, Se, and Zn) in three different medicinal plant species including the dry samples and teas from *B. forficata*, *E. indica*, and *O. stamineus* using the technique of inductively coupled plasma optical emission spectrometry (ICP OES) and to assess the hazard indices (HI) due to ingestions of infusions of plants in water. The results on dry samples were discussed by comparing with studies reported in the literature as well as regulatory limits of the WHO for metals in medicinal plant and the permissible limit set by the Food and Agriculture Organization of the United Nations (FAO/WHO) for metals in edible plant.

## 2. Materials and Methods

### 2.1. Sample Collection

A total of 15 samples for each species of commercially available medicinal plants were randomly purchased from the company “Cha & Cia Produtos Naturais” in Brazil during the period 2019-2020. The plant samples were commercially available in the plastic containers with pieces of leaves of *O. stamineus*, and leaves of *B. forficata*; each plastic container contains 100 g of the herb's raw material. On the other hand, the *E. indica* plants (10 plastic containers with leaves) were acquired through the purchase of Companhia de Produtos Naturais Sítio Menino Vaqueiro, Itapipoca, Ceará, Brazil.

### 2.2. Digestion Procedure for Dry Plants: *O. stamineus*, *B. forficata*, and *E. indica*

Plant parts and species for analysis were leaves of the *O. stamineus*, leaves of the *B. forficata*, and leaves of the *E. indica*. All samples were subjected to a drying process in a hot oven at 40°C for 12 hours. An amount of the 100 g of each dried sample was crushed separately with a portable stainless steel electric grinder to obtain a very fine powder (Thermomix, Brazil) and then sieved (stainless steel sieve, 200 *μ*m granulometry). Approximately 0.30 g of each sample was placed in a teflon microwave digestion tube and added 3.0 mL of HNO_3_ (65%, Merck, Darmstadt, Germany), 1.0 mL of high-purity water (18 M*Ω* cm, Milli-Q, Millipore, Bedford, MA, USA), and 2.0 mL of H_2_O_2_ (35%, Merck, Darmstadt, Germany). The digestion procedure was performed in triplicates. All tubes with samples of each plant were placed in the microwave digestion system (BERGHOF Products + Instruments GmbH - Speedwave 4 - Microwave Digestion System), according to the digestion program as depicted in Table 1.

### 2.3. Digestion Procedures for Plant Tea: *O. stamineus*, *B. forficata*, and *E. indica*

According to the labeling of plastic packaging of medicinal plants sold by companies in Brazil, the infusion process happens by adding two, three, or four tablespoons of the plant in a liter of water. As an experimental quality control to the methodologies, we consider 1.2 grams of sample powder equivalent to four tablespoons.

The infusions of each plant were performed as follows: i.e., to 1.2 g of each sample powder was added 30 mL of ultrapure water at 90°C and then allowed to stand for 15 min. Subsequently, the hot plate was turned off and the beaker was capped for 10 minutes to cool. After cooling the tea from each plant, it was filtered to remove impurities. Next, 8 mL aliquots of filtered tea from each plant were collected and each sample was placed in a teflon microwave digestion tube and added 1.0 mL of HNO_3_ (65% Merck, Darmstadt, Germany), and 0.5 mL of H_2_O_2_ (30%, Merck, Darmstadt, Germany). The microwave digestion system was programmed according to Table 2. After the microwave digestion stage, the samples were diluted to final volume of 10 mL with high-purity water. Blanks were prepared in each sample batch. All experiments were performed in triplicate.

### 2.4. Stock Solutions, Concentration Range of Calibration Curves

Multielementary standard stock solutions of 100 mg/L of Al, Co, Ca, Cr, Cu, Fe, K, Mg, Mn, Na, Ni, P, Se, and Zn (Specsol, São Paulo, Brazil) were utilized. The calibration standards were prepared by diluting the stock multielemental solutions as the following: 0.01, 0.025, 0.05, 0.1, 0.25, 0.5, 1.0, 2.0, and 4.0 ppm.

### 2.5. Elementary Analysis Using ICP OES

The content of macro- and microelements in the leaves of the *E. Indica* plant, leaves of *O. stamineus*, and leaves of *B. forficata*, as well as their teas, was quantified using inductively coupled plasma optical emission spectrometry (ICP OES, Thermo Fisher Scientific, Bremen, Germany, model iCAP 6300 Duo). The instrumental parameters of ICP OES are shown in Table 3.

According to the IUPAC method [39], the limit of detection (LOD) and limit of quantification were determined using

(1) $$\begin{array}{c} \text{LOD}=\frac{3\;\times \;Sb}{m}, \end{array}$$

(2) $$\begin{array}{c} \text{LOQ}=\;\frac{10\;\times Sb}{m}. \end{array}$$

In Equations (1) and (2), *Sb* represents the standard deviation of ten replicates (*n* = 10), where LOD is 3 times the standard deviation of blank absorbance signal (*Sb*) divided by the slope (*m*) of the calibration curve, whereas the limit of quantification (LOQ) was defined as 10 × *Sb* divided by the slope (*m*) of the calibration curve. The LOD and LOQ values for each element and correlation coefficient (*R*^2^) of the calibration curve are shown in Table 4. Detection limits ranged from 0.002 to 0.02 mg/L. The quantification limits ranged from 0.003 to 0.1 mg/L.

To ensure the precision of the experiment to the quantification of elements in samples, the Standard Reference Material (SRM) 1575a in Pine Needles (*Pinus taeda*) was used for validation of the method (Table 5). The recoveries range from 91.83% to 103.53%, which proved that this method was accurate and precise.

### 2.6. Hazard Quotient (HQ) Method

The potential health risks of macro- and microelement consumption through teas of the plants were assessed based on the target hazard quotient method (HQ) developed by the United States Environmental Agency (USEPA) [40]. The HQ is given by the following equation:

(3) $$\begin{array}{c} \text{HQ}=\frac{\left(\text{EF}\times \text{DE}\times \text{IR}\times C\right)}{\left(\text{RfD}\times W\times \text{TE}\right)}, \end{array}$$

where EF is the exposure frequency (EF = 90 days/year and EF = 365 days/year). DE is the exposure duration (70 years); IR is the tea ingestion rate, that is, the consumption values of tea for adults were considered 1.20 g/day. *C* is the concentration of macro- or microelements in tea of the plants quantified by ICP OES (mg/kg). RfD is the oral reference dose (*μ*g/g/day), established by the USEPA (Table 6) [41]. *W* is the mean body weight (70 kg); TE is the averaging time, noncarcinogens (365 days/year × ED = 25550 days, assuming 70 years).

Since plants contain more than one heavy metal, the hazard index (HI) is the sum of the hazard quotients, as described in

(4) $$\begin{array}{c} \text{HI}=\sum \text{Q}\text{R}=\sum {\text{QR}}_{\text{macro}}+\sum {\text{QR}}_{\text{micro}}. \end{array}$$

An HI > 1 indicates that the consumer population faces a health risk.

### 2.7. Statistical Analysis

One-way analysis of variance (ANOVA) and Tukey's post hoc multiple comparison tests were used to test for significant differences in the levels of elements in dry plants. In addition, ANOVA was used to test for significant difference concentrations of tea made with four tablespoons of each plant.

## 3. Results and Discussion

### 3.1. Concentrations of Metals and Nonmetals in Dried Plants

The results of the total concentrations of the elements studied in the powdered leaves of these plants (Table 7) demonstrate the presence of chemical elements such as K, Na, Mg, Fe, Al, and Mn in higher concentrations, and Zn and Cu in smaller quantities. The results also showed that some plant species have nonmetal concentration of P and Se. In all medicinal plants, the element Co is below the detection limit. The statistical tests indicate that the mean difference of the chemical elements quantified in dry plants is not significant at the 0.05 level. In discussing our results, we made it clear that the quantified elements in plants cannot be present as an effect of the individual preferences of the plants studied but can be related to contaminants such as pesticides in soil, water, and air, chemical toxins, geography, geochemical characteristic soil, transport, and storage conditions [42]. Accordingly, our results agreed with Ref. [10] in which K, Mg, Mn, Al, and Fe are the highest element in dried plants. In addition, zinc is the metal that is most commonly found in medicinal plants, that is, Zn is present in 88 medicinal plant species studied. Other elements as Fe and Cu were found in 87 of the plant species and Se were found in 21 species, respectively [43].

In the dry powdered leaves of *E. indica* and dry powdered leaves of *B. fortificata*, there was no detection of nickel (<LOD). The concentration of Se in dry powdered leaves of *E. indica* and dry powdered leaves of *O. stamineus* is below the detection limit.

In the present study (Table 7), the concentration of macroelements in the powdered leaves of *E. indica* decreases in the following order: K > Na > Mg > P, while the concentration of microelements decreases Fe > Al > Mn > Zn > Cu > Ni. On the other hand, the concentrations of macroelements in the dry powdered leaves of *O. stamineus* decrease in the following order: K > Mg > Na > P; and microelements: Fe > Mn > Al > Zn > Cu. In addition, analysis of the experimental results revealed in Table 6 that the concentration of macroelements in the dry powdered leaves of *B. fortificata* was K > Mg > Na > P, while for the microelements, they were as follows: Fe > Mn > Al > Cu > Zn = Se.

For a better understanding of our results, the concentration of each macro- and microelements and their variations in the analysis of different plants were compared with the regulatory limits of the WHO (2005) for metals in medicinal plant [44], and permissible limits set by the FAO/WHO for metals in edible plants [45]. The results of the comparisons are presented in the text below according to the order of each element shown in Table 7.

In Table 7, the powdered leaves of *E. indica* have higher K concentration that is 96886.2 ± 1997.0 mg/kg than the other, while *O. stamineus* was 79071.9 ± 1020.0 mg/kg and for *B. fortificata* was 80680.3 ± 2260.0 mg/kg. The regulatory limits of the WHO (2005) for medicinal plant have not been established yet for the K [44]. In addition, the permissible limit set by the FAO/WHO for K in edible plants has not been established yet [45]. To date, there are no reports of toxicity of potassium from consumption in food. Nevertheless, the case reports have shown that excessive potassium supplement intake can lead to adverse events and intoxication death [46]. The dietary intake levels have been associated with incident diabetes. In fact, according to studies, lower levels of potassium have been found to be associated with a higher risk of diabetes [47].

In studied dry powdered leaves, Mg concentration was 18399.9 ± 499.0 mg/kg in *E. indica*, 17594.25 ± 48.45 mg/kg in *O. stamineus*, and 17381.0 ± 54.0 mg/kg in *B. fortificata* (Table 7). The permissible level set by the FAO/WHO for Mg in edible plants is 200 mg/kg. The limits of the WHO for medicinal plant have not been established yet for the Mg. After comparison, limit in the studied medicinal plants with those proposed by the FAO/WHO, it is found that all plants have Mg concentrations above this limit [45]. Magnesium toxicity is rare in the general population; however, magnesium in high concentrations can cause severe toxic effects in human patients [48]. Intracellular Mg plays a key role in regulating insulin action, insulin-mediated glucose uptake, and vascular tone [49].

As can be seen in Table 7, the concentration of Na in *E. indica* leaves, *O. stamineus* leaves, and *B. fortificata* leaves was found to be 18747.1 ± 280.5 mg/kg, 8343.7 ± 182.8 mg/kg, and 7961.0 ± 90.7 mg/kg, respectively. There are no established limits for Na in medicinal plants, as well as there are no permissible limit set by the FAO/WHO in edible plants. According to the WHO (2012), sodium is found naturally in a variety of foods, such as vegetables and fresh; it is estimated to be 10 mg/100 g [50]. After comparison, the Na values in the studied medicinal plants in mg/100 g (Table 7) with those estimated by the WHO, it was found that the leaves of *E. indica*, leaves of *O. stamineus*, and leaves of *B. fortificata* contain Na above that value. Diets higher in sodium are associated with an increased risk of developing high blood pressure [51]. Thus, the ingestion of medicinal plants with high sodium content should be avoided, as patients with type 1 diabetes show a significant increase in blood pressure to salt in the salt-rich diet [52].

The content of P in powdered leaves of *E. indica* was 47.95 ± 0.35 mg/kg. In addition, the amount of P concentration in powdered leaves of *O. stamineus* was 46.39 ± 0.07 mg/kg and 45.64 ± 0.22 mg/kg in the powdered leaves of *B*. *fortificata* (Table 7). The limits of the WHO for medicinal plant have not been established yet for the P. The permissible limit set by the FAO/WHO for P in edible plants has not been established yet. Phosphorus intoxication from excessive consumption in food is not known; however, phosphorus is toxic to humans [53]. The serum level of phosphorus is obviously decreased in patients with type 2 diabetes [54], so foods and medicinal plants rich in P can help control the disturbance in phosphorus metabolism.

The concentration of Al in the powdered leaves of *E. indica* was 1220.4 ± 30.6 mg/kg, while that for powdered leaves of *O. stamineus* was 460.8 ± 24.1 mg/kg and for powdered leaves of *B. fortificata* was 452.7 ± 17.5 mg/kg (Table 7). There are no limits yet established by the FAO/WHO for Al in edible plants. For medicinal plants, the WHO limits are not yet established for Al. However, according to the consumption analysis presented in 1989 by the FAO/WHO Committee of Experts for food additives, the daily intake of aluminum in adults is 6-14 mg/kg [55]. After comparison, Al content in the studied medicinal plants with those proposed by the FAO/WHO for additives, it is found that all plants in Table 7 have Al above this limit. Studies conducted in Wuhan, China, showed that aluminum levels were associated with a risk of gestational diabetes [56].

The concentration of Fe in the dry powdered leaves of the *E. indica*, *O. stamineus*, and *B. fortificata* was 1605.1 ± 29.2 mg/kg, 657.8 ± 4.9 mg/kg, and 652.53 ± 5.85 mg/kg (Table 7). FAO/WHO permissible limits for iron (Fe) specifically in edible plants are 20 mg/kg [45]. After comparison, Fe levels in leaves of *E. indica*, *O. stamineus*, and *B. fortificata* with those proposed by the FAO/WHO, it is found that these leaves of plants contain iron above this limit. However, for medicinal plants, the WHO (2005) limit has not yet been established for Fe. Iron has an essential role in numerous metabolic pathways in the body; however, iron overload is a risk factor for diabetes [57].

Among the investigated medicinal plants, dry powdered leaves of *E. indica* exhibited higher Zn concentration, that is, 51.48 ± 0.65 mg/kg, and *O. stamineus* and *B. fortificata* possess minimum concentration of Zn that is 16.57 ± 0.14 mg/kg and 16.44 ± 0.12 mg/kg. The permissible limit set by the FAO/WHO (1984) for zinc is 27.4 mg/kg in edible plants [44], while the permissible WHO (2005) limit for Zn in medicinal plants is 50 mg/kg [45]. After comparison, metal limits in the medicinal plant studied with those proposed by the FAO/WHO and WHO (2005), it is found leaves of *E. indica* have Zn above the limit set by the FAO/WHO and WHO, with exception of the Zn content in *O. stamineus* and *B. fortificata* plants which are below these limits. Although zinc is considered relatively nontoxic to humans, only exposure to high doses has toxic effects, making acute zinc intoxication a rare event [58]. Previous studies have been shown that supplementation of zinc to type 2 diabetes patients improves the symptoms of diabetes [59–61].

The concentration of manganese (Mn) was 479.58 ± 8.04 mg/kg in dry powdered leaves of *E. indica*, followed by dry powdered leaves of *O. stamineus* which is 470.90 ± 4.03 mg/kg and in *B. fortificata* was 462.9 ± 0.1 mg/kg. The permissible limit set by the FAO/WHO for manganese (Mn) is 2.0 mg/kg in edible plants [45]. Therefore, the manganese content of dry powdered leaves of *E. indica*, *O. stamineus*, and *B. fortificata* shown in Table 7 is above the permissible levels. Until 2019, there is no permissible limit for Mn by the WHO in medicinal plants. Mn is both a toxic and an essential trace element for human health. According to the paper published by Wang et al. [62], several studies have reported that appropriate serum manganese levels may help prevent and control prediabetes and diabetes [62–64].

The concentration of Cu in dry powdered leaves of *E. indica* was 32.96 ± 0.84 mg/kg, 22.42 ± 0.49 mg/kg in dry powdered leaves of *O. stamineus*, and 22.20 ± 0.32 mg/kg in dry powdered leaves of *B. fortificata* (Table 7). The permissible limit for Cu set by the FAO/WHO in edible plants is 3 mg/kg [45]. For medicinal plants, the WHO (2005) limits were not yet been established for Cu. Permissible limits for Cu set by China and Singapore for medicinal plants were 20 and 150 mg/kg, respectively [65]. Thus, the Cu concentrations obtained in the present study are above the values established by the FAO/WHO for edible plants and within the values allowed by China and Singapore for medicinal plants. Copper plays a vital role in various metabolic processes in humans; however, it can pose risks to human health with high exposure and create oxidative stress, which is a factor in the progression of type 2 diabetes mellitus [66].

The presence of Ni takes place only in the leaves of *E. indica*, that is, 3.35 ± 0.07 mg/kg (Table 7). The permissible limit for nickel set by the FAO/WHO (1984) in edible plants is 1.63 mg/kg, and the permissible limits for medicinal plants have yet not been set by the WHO (2005). On comparing the metal limit in the *E. indica* plant with those proposed by the FAO/WHO [45], it was found that leaves of plant have nickel above this permissible limit. Nickel toxicity and its compounds in humans are not of very common occurrence. The absorption by the body is affected by the type of food ingested and the prior presence of food in the stomach [67]. According to studies in China, the increased urinary nickel concentration is associated with an elevated prevalence of type 2 diabetes [68].

In Table 7, the concentration of Se in the dry powdered leaves of *B. fortificata* was 16.44 ± 0.12 mg/kg. There are no limits established yet by the FAO/WHO for Se in edible plants. The permissible WHO (2005) limits for Se in medicinal plants have not yet been set. Selenium toxicity due to overdose is rare due to intake foods, but selenium can have an adverse effect on health at high exposure [69]. Selenium used as a dietary supplement has its dietary benefits proven in patients with type II diabetes [70].

The presence of elements such as K, Mg, Na, P, Al, Fe, Zn Mn, Cu, Ni, and Se in the leaves of *E. indica*, leaves of *O. stamineus*, and leaves *of B. fortificata* shows that such plants may have a possible action benefits when used in popular medicine. Other studies corroborate our proposition, that is, the high concentration of K, Mn, Cu, and Zn in several antidiabetic medicinal plants has been made responsible for the stimulation of insulin action [10]. However, the prescription of these plants in capsules for patients should be carried out with caution, since long-term ingestion or in large quantities can interfere with the absorption of other elements and cause toxicity as shown above.

### 3.2. Mineral Contents of Herbal Infusions

The results of the mineral content of medicinal plants presented in Subsection 3.2.1 will later be used to perform in Subsection 3.2.2 the risk calculations of the potential health risks of the consumption of teas from the plants evaluated based on the method of the target risk quotient.

#### 3.2.1. Infusion Tea of *E. indica*, *O. stamineus*, and *B. fortificata*

The results of the total concentration of studied metal and nonmetal in infusion tea of *E. indica*, *O. stamineus*, and *B. fortificata* (Table 8) show the highest levels of metals and nonmetal, especially Na, P, and Mn, to a lesser extent Zn, Fe, Cu, Ni, Cu, Co, and Se. Besides that, there is no statistically significant difference between the mean of concentration of elements in the teas of each plant (*p* > 0.05). Our results were in agreement with several published studies, in which Mn was found in greater quantity in 18 types of infused tea samples [71], as well as Na, Fe, P, Cu, Zn, and Se in infusions of herbs widely used as sedatives [72], and Ni and Co in herbal tea infusion [73]. The concentration of elements such as Mg, Al, Fe, and Co in some of the tea leaves is below the limit of detection (Table 8). The concentration of K in plants is above the values of the calibration curves.

In tea prepared with 1.20 g of powdered leaves of *E. indica* (Table 8), the macroelement concentration as Na was 9.81 ± 0.61 mg/kg, and P concentration was 27.7 ± 0.8 mg/kg, while that for the microelements, the order is as follows: Mn (2.08 ± 0.06 mg/kg) > Zn (0.51 ± 0.03 mg/kg) > Fe (0.19 ± 0.05 mg/kg) > Ni (0.019 ± 0.001 mg/kg) > Se (0.129 ± 0.004 mg/kg) > Cu (0.014 ± 0,001 mg/kg) > Co (0.003 ± 0.0001 mg/kg). The percentages of mass transfer from the 1.20 g of powdered leaves of *E. indica* to infusion solution were Na: 0.523%, P: 57.831%, Fe: 0.011%, Zn: 0.990%, Mn: 0.433%, Cu: 0.042%, and Ni: 0.567%.

According to Table 8, in teas prepared with 1.2 g of powdered dry leaves of *O. stamineus*, the following macroelements were obtained: Na (12.30 ± 0.56 mg/kg) and P (5.55 ± 0.12 mg/kg). The concentration of microelements was found as Mn (0.77 ± 0.01 mg/kg) > Zn (0.11 ± 0.01 mg/kg) > Fe (0.03 ± 0.05 mg/kg) > Se (0.015 ± 0.001 mg/kg) > Cu (0.011 ± 0.002 mg/kg) > Ni (0.010 ± 0.002 mg/kg). The percentages of mass transfer from the 1.20 g powdered leaves of *O. stamineus* to the infusion solution were Na: 0.1774%, P: 11.963%, Fe: 0.0045% Zn: 0.6638%, Mn: 0.1643%, and Cu: 0.049%.

For tea prepared with 1.20 grams of powdered dry leaves of the *B*. *fortificata* (Table 8), the macroelement concentrations such as Na and P were 14.29 ± 0.99 mg/kg and 144.43 ± 0.31 mg/kg, respectively, with the following microelement concentrations: Mn (0.667 ± 0.009 mg/kg) > Zn (0.396 ± 0.007 mg/kg) > Cu (0.187 ± 0.004 mg/kg) > Fe (0.084 ± 0.006 mg/kg) > Ni (0.038 ± 0.0005 mg/kg) > Co (0.005 ± 0.0001 mg/kg). The percentages of mass transfer from the 1.20 g powdered leaves of *B. fortificata* to the infusion solution were Na: 0.178%, P: 316.454%, Fe: 0.012%, Zn: 2.408%, Mn: 0.144%, Cu: 0.842%, and Se: 0.358%. As we can see above, the transfer of metals from the dried plant during infusion depends on the species, age of leaf, geographic location, etc. [74].

#### 3.2.2. Risk Assessment for Health

The hazard quotients (HQs) and hazard index (HI) for Fe, Zn, Mn, Co, Cu, Ni, and Se through consumption of plant leaf tea (*E. Indica, O. stamineus*, and *B. forficata*) for an exposure frequency (EF) of 90 days/year and 365 days/year and ingestion rate (IR) of 1.20 g/day are shown in Table 9. There are no oral reference doses (RfD) for metals as K, Mg, and Na and nonmetal as P.

In this study, the hazard index (HI) for metal from individual plants was less than one, which is considered as safe for human consumption at age 70 years. In fact, even considering an exposure frequency of 365 days/year, and an ingestion rate of 1.20 g/day, all HI values do not exceed 1. The results presented for HI in Table 9 are in agreement with those obtained in Iran for other species of medicinal plants, that is, for an exposure frequency of 365 days/year and exposure duration of 70 years, the HI values are less than 1 [75].

## 4. Conclusions

The study showed that dry samples and teas from B. forficata, *E. indica*, and *O. stamineus* used to treat diabetes contained elements such as K, Mg, Na, P, Al, Fe, Zn, Mn, Cu, Ni, and Se.

All dry plants have concentrations of Mg, Al, Fe, Mn, Ni, and Cu above the limit permissible level set by the FAO/WHO in edible plants and Na above the value estimated by the WHO (2012). The concentration of Zn in the leaves of *E. indica* is above the limit established by the FAO/WHO and WHO. The regulatory limits of the WHO for plants have not been established yet for the K, P, and Se. However, plants contain significant amounts of K, P, and Se. Thus, very low concentrations of some metals can be toxic and cause serious health problems when ingested through capsules prepared by enclosing a plant powder, or homogeneous dry extract powder or granules with excipients in a suitable capsule base such as gelatin.

The presence of selenium and other chemical elements in dry plants and its infusion that has a positive character due to human clinical trials found improvements in measurements of glucose, insulin, etc. Based on the calculated magnitude of the health risk assessment per dosage (hazard index < 1), the tea from the medicinal plants investigated in this study might not pose any risk to human health. However, the prescription of this plant tea for the treatment of diabetes should be treated with caution.

The new information obtained in the present study on elemental compositions *B. forficata*, *E. indica*, and *O. stamineus* used to treat diabetes will be useful in deciding the dosage of the drugs prepared from the plants. However, in order to develop a stronger basis for appreciating the healing effects of these plants, there is a need to study animal models and to monitor people who use these plants.

According to studies, diabetes seems prevalent when Zn, Se, and Cu are deficient; thus, physiological effects of elements (K, Mg, P, Fe, Zn, Cu, Se, etc.) in low concentrations can act in the body as a medicine (producing a sanogenetic effect), whereas sodium, aluminum, nickel, potassium, iron, magnesium, and some other elements in high concentrations can cause severe health effects.

## Figures and Tables

**Table 1:** Operating conditions for the microwave-assisted acid digestion system: dry plant.

Parameter	Stage		
	1	2	3
Power (W)	1120	1120	0
Temperature (°C)	160	190	50
Hold time (min)	5	10	10
Pressure (bar)	35	35	0

**Table 2:** Operating conditions for the microwave-assisted acid digestion system for plant tea.

Parameter	Stage		
	1	2	3
Power/W	1160	1305	0
Temperature/°C	160	190	50
Hold time/min	5	10	10
Pressure/bar	40	40	0

**Table 3:** ICP OES instrumental parameters.

Parameter	Setting
RF power (W)	1250
Sample flow (L min^−1^)	0.35
Replicates	3
Plasma flow rate (L min^−1^)	12
Integration time (s)	5
Stabilization time (s) Nebulization pressure(psi)	20
	30
Plasma view	Axial
Analytes/*λ*	Al 396.100 nm, Co 228.616 nm, Cu 324.754 nm, Fe 259.940 nm, K 766.490 nm, Mg 279.553 nm, Mn 257.610 nm, Na 588.995 nm, Ni 221.647 nm, P 214.914 nm, Se 196.00 nm, Zn 213.856 nm

**Table 4:** Analytical characteristics of the ICP OES method: limit of detection (LODs), limit of quantification (LOQs), and correlation coefficient (*R*^2^*).*

Elements	LOD (mg/L)	LOQ (mg/L)	Correlation coefficient/*R*^2^
Al	0.02	0.006	0.9998
Co	0.002	0.008	0.9998
Cu	0.002	0.005	0.9998
Fe	0.002	0.006	0.9998
K	0.002	0.006	0.9999
Mg	0.009	0.03	0.9990
Mn	0.002	0.005	0.9999
Na	0.04	0.1	0.9999
Ni	0.002	0.007	0.9999
P	0.02	0.06	0.9997
Se	0.003	0.009	0.9998
Zn	0.001	0.003	0.9985

**Table 5:** Determined and certified values of elements in SRM 1575a (*Pine Needles*), *n* = 3.

Elements	Determined value (mg/kg) ICP OES	Certificated value (mg/kg) SRM 1575a	Recovery (%)
Al	560.2 ± 50	580 ± 30	96.55
Co	0.232 ± 0.005	0.230 ± 0.004	100.86
Cu	2.9 ± 0.22	2.8 ± 0.2	103.57
Fe	43.6 ± 3.0	46 ± 2.0	94.78
K	3829.48 ± 404.76	4170 ± 70	91.83
Mg	982.50 ± 30.20	1060 ± 160	92.68
Mn	451.70 ± 46.1	488 ± 12	95.56
Na	62.89 ± 3.55	63 ± 1	99.82
Ni	1.72 ± 0.040	1.47 ± 0.1	117
P	1059.06 ± 3.10	1070 ± 80	98.97
Se	0.090 ± 0.001	0.099 ± 0.004	90.9
Zn	35 ± 0.2	38 ± 2	92.1

**Table 6:** Oral reference dose (RfD) for metals.

Elements	RfD (mg/kg/day)
Al	1.0
Co	0.0003
Cu	0.04
Fe	0.70
K	NS
Mg	NS
Mn	0.14
Na	NS
Ni	0.02
P	NS
Se	0.005
Zn	0.30

NS = not specified.

**Table 7:** Concentrations of macro- and microelements in dry powdered leaves of *E. indica*, *O. stamineus*, and *B. fortificata* (mean ± SD).

	Dry powdered leaves of *E. indica*	Dry powdered leaves of *O. stamineus*	Dry powdered leaves of *B. fortificata*
Macroelements	(mg kg^−1^)	(mg kg^−1^)	(mg kg^−1^)
K	96886.2 ± 1997.0	79071.9 ± 1020.0	80680.3 ± 260.0
Mg	18399.9 ± 499.0	17594.25 ± 48.45	17381.0 ± 54.0
Na	18747.1 ± 280.5	8343.7 ± 182.8	7961.0 ± 90.7
P	47.95 ± 0.35	46.39 ± 0.07	45.64 ± 0.22
Microelements			
Al	1220.4 ± 30.6	460.8 ± 24.1	452.7 ± 17.5
Fe	1605.1 ± 29.2	657.8 ± 4.9	652.53 ± 5.85
Zn	51.48 ± 0.65	16.57 ± 0.14	16.44 ± 0.12
Mn	479.58 ± 8.04	470.90 ± 4.03	462.9 ± 0.1
Co	<LOQ	<LOQ	<LOQ
Cu	32.96 ± 0.84	22.42 ± 0.49	22.20 ± 0.32
Ni	3.35 ± 0.07	<LOQ	<LOD
Se	<LOQ	<LOQ	16.44 ± 0.12

Below the limit of detection (<LOD).

**Table 8:** Concentration of macro- and microelements in leaf tea for four tablespoons (mg/kg) determined by ICP OES.

	Plants		
	*E. indica*	*O. stamineus*	*B. fortificata*
Macroelements			
K	ND	ND	ND
Mg	<LOQ	<LOQ	<LOQ
Na	9.81 ± 0.61	12.30 ± 0.56	14.29 ± 0.99
P	27.7 ± 0.8	5.55 ± 0.12	144.43 ± 0.31
Microelements			
Al	<LOQ	<LOQ	<LOQ
Fe	0.19 ± 0.05	0.03 ± 0.05	0.084 ± 0.006
Zn	0.51 ± 0.03	0.11 ± 0.01	0.396 ± 0.007
Mn	2.08 ± 0.06	0.77 ± 0.01	0.667 ± 0.009
Co	0.003 ± 0.0001	<LOQ	0.005 ± 0.0001
Cu	0.014 ± 0.001	0.011 ± 0.002	0.187 ± 0.004
Ni	0.019 ± 0.001	0.010 ± 0.002	0.038 ± 0.0005
Se	0.129 ± 0.004	0.015 ± 0.001	0.059 ± 0.006

Below the limit of quantification (<LOD); ND: not determined.

**Table 9:** Hazard quotients (HQs) and hazard index (HI) for ingestion of macro- and microelements through consumption of tea from plant leaves for an exposure frequency (EF) of 90 days and 365 days and ingestion rate (IR) of 1.20 g/day.

Elements	Tea from the leaves (EF = 90/days)			Tea from the leaves (EF = 365/days)		
	*E. Indica*	*O. stamineus*	*B. forficata*	*E. Indica*	*O. stamineus*	*B. forficata*
	HQ	HQ	HQ	HQ	HQ	HQ
K	NQ	NQ	NQ	NQ	NQ	NQ
Mg	NQ	NQ	NQ	NQ	NQ	NQ
Na	NS	NS	NS	NS	NS	NS
P	NS	NS	NS	NS	NS	NS
Al	NQ	NQ	NQ	NQ	NQ	NQ
Fe	1.1413 × 10^−6^	1.811 × 10^−7^	5.0724 × 10^−7^	4.628 × 10^−6^	7.346 × 10^−7^	2.0571 × 10^−6^
Zn	7.2141 × 10^−6^	1.549 × 10^−6^	5.5796 × 10^−6^	2.925 × 10^−5^	6.285 × 10^−6^	2.2628 × 10^−5^
Mn	6.2892 × 10^−5^	2.336 × 10^−5^	2.0138 × 10^−5^	2.5506 × 10^−4^	9.477 × 10^−5^	8.1673 × 10^−5^
Co	4.2270 × 10^−5^	NQ	7.0450 × 10^−5^	1.7143 × 10^−4^	NQ	2.8571 × 10^−4^
Cu	1.4794 × 10^−6^	1.162 × 10^−6^	1.9761 × 10^−5^	6.0 × 10^−6^	4.714 × 10^−6^	8.0143 × 10^−5^
Ni	4.0156 × 10^−6^	2.1135 × 10^−6^	8.0313 × 10^−6^	1.6286 × 10^−5^	8.571 × 10^−6^	3.2571 × 10^−5^
Se	1.0905 × 10^−4^	1.2681 × 10^−5^	4.9878 × 10^−5^	4.4228 × 10^−4^	5.142 × 10^−5^	2.0228 × 10^−4^
HI^∗^	2.28 × 10^−4^	4.11 × 10^−5^	1.74 × 10^−4^	9.25 × 10^−4^	1.67 × 10^−4^	7.07 × 10^−4^

NQ = not quantified; NS = not specified.

## Data Availability

The data used to support the findings of this study are available from the corresponding author upon request.

## References

[1] American Diabetes Association (2008). Diagnosis and classification of diabetes mellitus. *Diabetes Care*.

[2] Saeedi P., Petersohn I., Salpea P. (2019). Global and regional diabetes prevalence estimates for 2019 and projections for 2030 and 2045: Results from the International Diabetes Federation Diabetes Atlas, 9th edition. *Diabetes Research and Clinical Practice*.

[3] World Health Organization/WHO (2018). Diabetes. https://www.who.int/news-room/fact-sheets/detail/diabetes.

[4] World Health Organization (2016). Diabetes. https://www.who.int/health-topics/diabetes#tab=tab_1.

[5] Pandey A., Tripathi P., Pandey R., Srivatava R., Goswami S. (2011). Alternative therapies useful in the management of diabetes: a systematic review. *Journal of Pharmacy & Bioallied Sciences*.

[6] Babu P. A., Suneetha G., Boddepalli R. (2006). A database of 389 medicinal plants for diabetes. *Bioinformation*.

[7] Kooti W., Farokhipour M., Asadzadeh Z., Ashtary-Larky D., Asadi-Samani M. (2016). The role of medicinal plants in the treatment of diabetes: a systematic review. *Electronic Physician*.

[8] Singh A., Zhao K. (2017). Herb-Drug Interactions of Commonly Used Chinese Medicinal Herbs. *International Review of Neurobiology*.

[9] Singh R., Kaur N., Kishore L., Gupta G. K. (2013). Management of diabetic complications: a chemical constituents based approach. *Journal of Ethnopharmacology*.

[10] Raju G. J. N., Sarita P., Murty G. A. V. R. (2006). Estimation of trace elements in some anti-diabetic medicinal plants using PIXE technique. *Applied Radiation and Isotopes*.

[11] Mertz W. (1993). Chromium in human nutrition: a review. *The Journal of Nutrition*.

[12] Rocha L. S., Arakaki D. G., Bogo D. (2019). Evaluation of level of essential elements and toxic metal in the medicinal plant Hymenaea martiana Hayne (Jatobá) used by mid-west population of Brazil. *The Scientific World Journal*.

[13] Rosa R. H., Fernandes M. R., Melo E. S. P. (2020). Determination of macro- and microelements in the inflorescences of banana tree using ICP OES: evaluation of the daily recommendations of intake for humans. *The Scientific World Journal*.

[14] Haidu D., Párkányi D., Moldovan R. I. (2017). Elemental characterization of Romanian crop medicinal plants by neutron activation analysis. *Journal of Analytical Methods in Chemistry*.

[15] Street R. A. (2012). Heavy metals in medicinal plant products -- An African perspective. *South African Journal of Botany*.

[16] Pohl P., Dzimitrowicz A., Jedryczko D., Szymczycha-Madeja A., Welna M., Jamroz P. (2015). The determination of elements in herbal teas and medicinal plant formulations and their tisanes. *Journal of Pharmaceutical and Biomedical Analysis*.

[17] World Health Organization/WHO (2011). Adverse health effects of heavy metals in children. Children's Health and the Environment. *WHO Training Package for the Health Sector*.

[18] Ortêncio W. B. (1997). *Medicina Popular Do Centro-Oeste, 2 edição*.

[19] Badgujar S. B. (2014). Evaluation of hemostatic activity of latex from three _Euphorbiaceae_ species. *Journal of Ethnopharmacology*.

[20] Coelho-Ferreira M. (2009). Medicinal knowledge and plant utilization in an Amazonian coastal community of Maruda, Para State (Brazil). *Journal of Ethnopharmacology*.

[21] Lino C. S., Diógenes J. P. L., Pereira B. A. (2004). Antidiabetic activity of *Bauhinia forficata* extracts in alloxan-diabetic rats. *Biological and Pharmaceutical Bulletin*.

[22] Franco R. R., Carvalho D. S., Moura F. B. R. (2018). Antioxidant and anti-glycation capacities of some medicinal plants and their potential inhibitory against digestive enzymes related to type 2 diabetes mellitus. *Ethnopharmacology*.

[23] Franco R. R., Alves V. H. M., Zabisky L. F. R. (2020). Antidiabetic potential of Bauhinia forficata Link leaves: a non-cytotoxic source of lipase and glycoside hydrolases inhibitors and molecules with antioxidant and antiglycation properties. *Biomedicine & Pharmacotherapy*.

[24] Davis S. N., Granner D. K., Hardman J. G., Limbird L. E., Molinoff P. B., Ruddon R. W., Gilman A. G. (1996). *Insulin, oral hypoglycemic agents, and the pharmacology of the endocrine pancreas*.

[25] Brondani G. E., Silva A. J. C., Araujo M. A., Grossi F., Wendling I., Carpanezzi A. (2008). A. Phosphorus nutrition in the growth of Bauhinia forficata L. seedlings. *Acta Scientiarum. Agronomy*.

[26] Okokon J. E., Odomena C. S., Effiong I., Obot J., Udobang J. A. (2010). Antiplasmodial and antidia beticactivities of eleusine indica. *International Journal of Drug Development & Research*.

[27] Penaloza E. M. C., Casanova L. M., Real I. C. R., Aguiar P. F., Costa S. S. (2018). Metabolite fingerprinting and profiling of the medicinal grass Eleusine indica based on HPLC-DAD, UPLC-DAD-MS/MS and NMR analyses. *Journal of the Brazilian Chemical Society*.

[28] Al-Zubairi A. S., Abdul A. B., Abdelwahab S. I., Peng C. Y., Mohan S., Elhassan M. M. (2011). Eleucine indica possesses antioxidant, antibacterial and cytotoxic properties. *Evidence-Based Complementary and Alternative Medicine*.

[29] Rojas-Sandoval J., Acevedo-Rodríguez P. (2014). Eleusine indica (goose grass). Retrieved October 30, 2016, from CABI. https://www.cabi.org/isc/datasheet/20675.

[30] Mohamed E. A. H., Yam M. F., Ang L. F., Mohamed A. J., Asmawi M. Z. (2013). Antidiabetic properties and mechanism of action of Orthosiphon stamineus Benth bioactive sub-fraction in streptozotocin-induced diabetic rats. *Journal of Acupuncture and Meridian Studies*.

[31] Lokman E. F., Saparuddin F., Omar M. H., Zulkapli A. (2019). _Orthosiphon stamineus_ as a potential antidiabetic drug in maternal hyperglycemia in streptozotocin-induced diabetic rats. *Integrative Medicine Research*.

[32] Gimbun J., Pang S. F., Yusoff M. M. (2019). Chapter 3.31- Orthosiphon stamineus (Java Tea). *Nonvitamin and Nonmineral Nutritional Supplements*.

[33] Bordbar L., Bhatt A., Subramaniam S., Lai-keng C. (2020). Necessity of medium nutrient modification for production of cell biomass yield and rosmarinic acid in the cell suspension cultures of Orthosiphon stamineus Benth. *International Journal of Medicinal and Aromatic Plants*.

[34] Gimbun J., Pang S. F., Yusoff M. M. (2019). Orthosiphon stamineus (java tea). *Nonvitamin and Nonmineral Nutritional Supplements*.

[35] Shanley P., Luz L. (2003). The impacts of forest degradation on medicinal plant use and implications for health care in Eastern Amazonia. *Bioscience*.

[36] Fennell C. W., Lindsey K. L., McGaw L. J., Sparg S. (2004). Assessing African medicinal plants for efficacy and safety: pharmacological screening and toxicology. *Journal of Ethnopharmacology*.

[37] Pandey M. M., Rastogi S., Rawat A. K. S. (2013). Indian traditional ayurvedic system of medicine and nutritional supplementation. *Evidence-based Complementary and Alternative Medicine*.

[38] Tschinkel P. F. S., Melo E. S. P., Pereira H. S. (2020). The hazardous level of heavy metals in different medicinal plants and their decoctions in water: a public health problem in Brazil. *BioMed Research International*.

[39] Long I. G., Winefordner J. D. (1983). Limit of detection: a closer look at the IUPAC definition. *Analytical Chemistry*.

[40] Pastorok P. (1987). Guidance manual for assessing human health risks from chemically contaminated fish and shellfish. *PTI Environmental Service’s submission to Battelle New England for EPA, Washington*.

[41] USEPA, (US Environmental Protection Agency) (2019). Regional Screening Level (RSL) Subchronic Toxicity Supporting Table November. https://www.epa.gov/risk/regional-screening-levels-rsls-generic-tables.

[42] Saad B., Azaizeh H., Abu-Hijleh G., Said O. (2006). Safety of traditional Arab herbal medicine. *Evidence–Based Complementary and Alternative Medicine*.

[43] Sarma H., Deka S., Deka H., Saikia R. R. Accumulation of heavy metals in selected medicinal plants. *Reviews of Environmental Contamination and Toxicology*.

[44] World Health Organization/WHO (2020). Quality Control Methods for Medicinal Plant Materials, Revised, Geneva. https://apps.who.int/iris/handle/10665/41986.

[45] Food and Agriculture Organization/Word Health Organization (FAOP/WHO) (1984). FAO/WHO, Food contaminants. *Codex Alimentarius*.

[46] Oria M., Harrison M., Stallings V. A., National Academies of Sciences, Engineering, and Medicine; Health and Medicine Division; Food and Nutrition Board (2019). Committee to Review the Dietary Reference Intakes for Sodium and Potassium. *Dietary Reference Intakes for Sodium and Potassium*.

[47] Chatterjee R., Yeh H. C., Edelman D., Brancati F. (2014). Potassium and risk of type 2 diabetes. *Expert Review of Endocrinology and Metabolism*.

[48] Ajib F. A., Childress J. M. (2020). Magnesium Toxicity. *StatPearls*.

[49] Barbagallo M., Dominguez L. J. (2015). Magnesium and type 2 diabetes. *World Journal of Diabetes*.

[50] WHO (2012). *Guideline: Sodium intake for adults and children*.

[51] Grillo A., Salvi L., Coruzzi P., Salvi P., Parati G. (2019). Sodium intake and hypertension. *Nutrients*.

[52] Wenstedt E. F. E., Rorije N. M. G., Engberink R. H. G. O. (2020). Effect of high-salt diet on blood pressure and body fluid composition in patients with type 1 diabetes: randomized controlled intervention trial. *BMJ Open Diabetes Research & Care*.

[53] Agency for Toxic Substances and Disease Registry (ATSDR) (1997). *Toxicological profile for white phosphorus. Atlanta*.

[54] Fang L., Li X. (2016). Level of serum phosphorus and adult type 2 diabetes mellitus. *Zhong Nan da Xue Xue Bao. Yi Xue Ban = Journal of Central South University. Medical Sciences*.

[55] Starska K. (1993). Aluminum in food. *Roczniki Panstwowego Zakladu Higieny*.

[56] Huang Y., Li X., Zhang W. (2020). Aluminum exposure and gestational diabetes mellitus: associations and potential mediation by n-6 polyunsaturated fatty acids. *Environmental Science & Technology*.

[57] Simcox J. A., McClain D. A. (2013). Iron and diabetes risk. *Cell Metabolism*.

[58] Plum L. M., Rink L., Haase H. (2010). The essential toxin: impact of zinc on human health. *International Journal of Environmental Research and Public Health*.

[59] Garg V. K., Gupta R., Goyal R. K. (1994). Hypozincemia in diabetes mellitus. *The Journal of the Association of Physicians of India*.

[60] Basaki M. (2012). Zinc, copper, iron and chromium concentrations in young patients with type 2 diabetes mellitus. *Biological Trace Element Research*.

[61] Jansen J. (2012). Disturbed zinc homeostasis in diabetic patients by in vitro and in vivo analysis of insulinomimetic activity of zinc. *The Journal of Nutritional Biochemistry*.

[62] Wang X., Zhang M., Lui G. (2016). Associations of serum manganese levels with prediabetes and diabetes among ≥60-year-old Chinese adults: a population-based cross-sectional analysis. *Nutrients*.

[63] Koh E. S., Kim S. J., Yoon H. E. (2014). Association of blood manganese level with diabetes and renal dysfunction: a cross-sectional study of the Korean general population. *BMC Endocrine Disorders*.

[64] Forte G., Bocca B., Peruzzu A. (2013). Blood metals concentration in type 1 and type 2 diabetics. *Biological Trace Element Research*.

[65] Ulla R., Khader J. A., Hussain I., AbdElsalam N. M., Talha M., Khan N. (2012). Investigation of macro and micro-nutrients in selected medicinal plants. *African Journal of Pharmacy and Pharmacology*.

[66] Bjørklund G., Dadar M., Pivina L., Doşa M. D., Semenova Y., Aaseth J. (2020). The role of zinc and copper in insulin resistance and diabetes mellitus. *Current Medicinal Chemistry*.

[67] Buxton S., Garman E., Heim K. E. (2019). Concise review of nickel human health toxicology and ecotoxicology. *Inorganics*.

[68] Liu G., Sun L., Pan A. (2015). Nickel exposure is associated with the prevalence of type 2 diabetes in Chinese adults. *International Journal of Epidemiology*.

[69] Antonyak H., Iskra R., Panas N., Lysiuk R., Malavolta M., Mocchegiani E. (2018). Selenium. *Trace Elements and Minerals in Health and Longevity. Healthy Ageing and Longevity*.

[70] Karalis D. T. (2019). The beneficiary role of selenium in type II diabetes: a longitudinal study. *Cureus*.

[71] Altıntıg E., Altundag H., Tuzen M. (2014). Determination of multi element levels in leaves and herbal teas from turkey by ICP-OES. *Bulletin of the Chemical Society of Ethiopia*.

[72] Petenatti M. E., Petenatti E. M., Del Vitto L. A. (2011). Evaluation of macro and microminerals in crude drugs and infusions of five herbs widely used as sedatives. *Revista Brasileira de Farmacognosia*.

[73] Eshmawy M. R., Ghuniem M., Khorshid M., Hammad G., Mahmoud S. M. (2019). Assessment of the potential health risk of heavy metal exposure from the consumption of herbal, black and green tea. *Biomedical Journal of Scientific & Technical Research*.

[74] Karak T., Bhagat R. M. (2010). Trace elements in tea leaves, made tea and tea infusion: a review. *Food Research International*.

[75] Kohzadi S., Shahmoradi B., Ghaderi E., Loqmani H., Maleki A. (2019). Concentration, source, and potential human health risk of heavy metals in the commonly consumed medicinal plants. *Biological Trace Element Research*.

